# Supplementary material for: Marine aquaculture as a source of propagules of invasive fouling species
Source: PeerJ. 2023 Jun 12;11:e15456. doi: 10.7717/peerj.15456 (PMC10269578; doi:10.7717/peerj.15456)
Supplement: Supplemental Information 1 — Current = species occurrences (white circles), Spalding et al. (2007) ecoregions (solid lines), and the states (abbreviations as in Fig. 1) at most risk of introduction and invasion, based on both connectivity and environment suitability (black = high risk, grey = medium risk, and light grey = low risk). RCP 6.0 = Future climate scenario in the year 2050. Environmental suitability maps were generated by the ensemble procedure of three types of Ecological Niche Models (MaxEnt, Support Vector Machine, and Random Forest). AB = Abrolhos bank, AR = Atol das Rocas, FN = Fernando de Noronha, SPSP = São Paulo São Pedro archipelago, TMV = Trindade – Martim Vaz archipelago. Ecoregions: 72 = Amazonia, 73 = Sao Pedro and Sao Paulo Islands, 74 = Fernando de Naronha and Atoll, das Rocas, 75 = Northeastern Brazil, 76 = Eastern Brazil, 77 = Trindade and Martin Vaz Islands, 180 = Southeastern Brazil, 181 = Rio Grande. [file peerj-11-15456-s001.pdf]

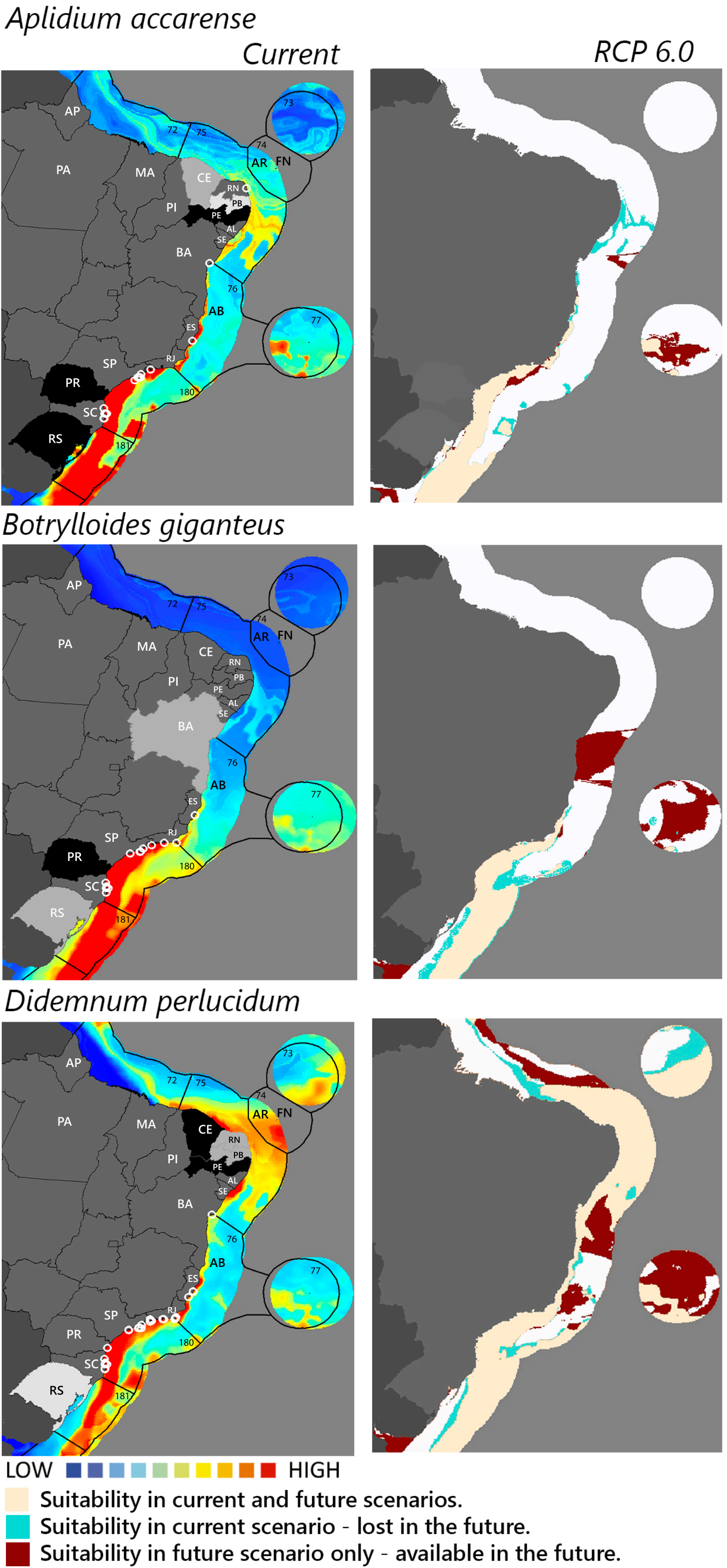
Aplidium accarense
Current
RCP 6.0
AP
PA
MA
CE
RN
PB
PI
PE
AL
SE
BA
ES
RJ
SP
PR
SC
RS
AR
FN
AB
72
73
74
75
76
77
180
181
Botrylloides giganteus
Didemnum perlucidum
LOW
HIGH
Suitability in current and future scenarios.
Suitability in current scenario - lost in the future.
Suitability in future scenario only - available in the future.
