# Supplementary material for: Marine aquaculture as a source of propagules of invasive fouling species
Source: PeerJ. 2023 Jun 12;11:e15456. doi: 10.7717/peerj.15456 (PMC10269578; doi:10.7717/peerj.15456)
Supplement: Supplemental Information 4 — The criteria was based on connectivity (total tonnage of goods in five years, 2015–2019) between Santa Catarina and destinations, and their environmental suitability estimated by the ensemble procedure of three types of Ecological Niche Models (MaxEnt, Support Vector Machine and Random Forest). [file peerj-11-15456-s004.pdf]

Supplementary material

Marine aquaculture as a source of propagules of invasive fouling species

Daniel M. Lins[1] and Rosana M. Rocha[2]

Table 1S. **Criteria used to define risk of species invasion.** The criteria was based on connectivity (total tonnage of goods in five years, 2015 – 2019) between Santa Catarina and destinations, and their environmental suitability estimated by the ensemble procedure of three types of Ecological Niche Models (MaxEnt, Support Vector Machine and Random Forest).

Connectivity	Environmental suitability	Risk of invasion
High	High or Intermediate	High
High	Low	Medium
Intermediate	High	High
Intermediate	Intermediate	Medium
Intermediate	Low	Low
Low	High	Medium
Low	Intermediate or Low	Low
