# Supplementary material for: Marine aquaculture as a source of propagules of invasive fouling species
Source: PeerJ. 2023 Jun 12;11:e15456. doi: 10.7717/peerj.15456 (PMC10269578; doi:10.7717/peerj.15456)
Supplement: Supplemental Information 5 — MaxEnt (MXS), Random Forrest (RDF), Support Vector Machine (SVM), and Ensemble (WMEA) evaluated using True Skill Statistics average (standard deviation in parenthesis). [file peerj-11-15456-s005.pdf]

Supplementary material

Marine aquaculture as a source of propagules of invasive fouling species

Daniel M. Lins[1] and Rosana M. Rocha[2]

Table 2S. Summary of predictive performance validated by random bootstrap partition of test occurrences (30% of N) of ten replications for the models: MaxEnt (MXS), Random Forrest (RDF), Support Vector Machine (SVM), and Ensemble (WMEA) evaluated using True Skill Statistics average (standard deviation in parenthesis).

**Species**	**N**	**MXS**	**RDF**	**SVM**	**WMEA**
Aplidium accarense	45	0.830 (0.032)	0.890 (0.039)	0.883 (0.044)	0.883 (0.041)
Botrylloides giganteus	20	0.693 (0.066)	0.896 (0.032)	0.883 (0.020)	0.853 (0.032)
Didemnum perlucidum	48	0.772 (0.045)	0.887 (0.088)	0.867 (0.096)	0.877 (0.083)
Styela plicata	121	0.843 (0.037)	0.944 (0.041)	0.894 (0.054)	0.932 (0.059)
Schizoporella errata	55	0.764 (0.082)	0.947 (0.019)	0.918 (0.037)	0.923 (0.018)
Watersipora subtorquata	48	0.821 (0.109)	0.965 (0.034)	0.972 (0.022)	0.954 (0.038)
Megabalanus coccopoma	80	0.814 (0.005)	0.954 (0.042)	0.914 (0.043)	0.906 (0.059)
Mytilus galloprovincialis	678	0.932 (0.015)	0.981 (0.005)	0.972 (0.005)	0.977 (0.009)
